# Anomalous opening of the common bile duct into the duodenal bulb: endoscopic treatment

**DOI:** 10.1186/1471-230X-7-26

**Published:** 2007-07-05

**Authors:** Selcuk Disibeyaz, Erkan Parlak, Bahattin Cicek, Cem Cengiz, Sedef O Kuran, Dilek Oguz, Hakan Güzel, Burhan Sahin

**Affiliations:** 1 Department of Gastroenterology, Turkiye Yüksek Ihtisas Hospital, Sihhiye, Ankara, Turkey; 2 Department of General Surgery, Diskapi Egitim ve Arastirma Hastanesi, Diskapi, Ankara, Turkey

## Abstract

**Background:**

Anomalous biliary opening especially the presence of the ampulla of Vater in the duodenal bulb is a very rare phenomenon. We report clinical implications, laboratory and ERCP findings and also therapeutic approaches in 53 cases.

**Methods:**

The data were collected from the records of 12.158 ERCP. The diagnosis was established as an anomalous opening of the common bile duct (CBD) into the duodenal bulb when there is an orifice observed in the bulb with the absence of a papillary structure at its normal localization and when the CBD is visualized by cholangiography through this orifice without evidence of any other opening.

**Results:**

A total of 53 cases were recruited. There was an obvious male preponderance (M/F: 49/4). Demographic data and ERCP findings were available for all, but clinical characteristics and laboratory findings could be obtained from 39 patients with full records. Thirty – seven of 39 cases had abdominal pain (95%) and 23 of them (59%) had cholangitis as well. Elevated AP and GGT were found in 97.4% (52/53). History of cholecystectomy was present in 64% of the cases, recurrent cholangitis in 26% and duodenal ulcer in 45%. Normal papilla was not observed in any of the patients and a cleft-like opening was evident instead. The CBD was hook shaped at the distal part that opens to the duodenal bulb. Pancreatic duct (PD) was opening separately into the bulb in all the cases when it was possible to visualize. Dilated CBD in ERCP was evident in 94% and the CBD stone was demonstrated in 51%. PD was dilated in four of 12 (33%) cases. None of them has a history of pancreatitis. Endoscopically, Papillary Balloon Dilatation instead of Sphincterotomy carried out in 19 of 27 patients (70%) with choledocholithiazis. Remaining eight patients had undergone surgery (30%). Clinical symptoms were resolved with medical treatment in 16(32%) patients with dilated CBD but no stone. Perforation and bleeding were occurred only in two patients, which stones extracted with sphincterotomy (each complication in 1 patient).

**Conclusion:**

The opening of the CBD into the duodenal bulb is a rare event that may be associated with biliary and gastric/duodenal diseases. To date, surgical treatment has been preferred. In our experience, sphincterotomy has a high risk since it may lead to bleeding and perforation by virtue of the fact that a true papillary structure is absent. However, we performed balloon dilatation of the orifice successfully without any serious complication and suggest this as a safe therapeutic modality.

## Background

Anatomic abnormalities associated with the opening of biliary system into the upper gastrointestinal tract have been increasingly recognized after more widely utilization of Endoscopic Retrograde Cholangiopancreatography (ERCP) in clinical practice. The common bile duct (CBD) normally goes next to the medial wall of the second part of the duodenum and opens into ampulla of Vateri. However, entrance into third or fourth part of the duodenum and less commonly into more proximal sites such as the stomach, pylorus or bulb have been described in the literature. [1-4]

Opening into the duodenal bulb has been indicated in a few case reports. The data about the clinical implications of this anatomic variation are very limited and the treatment usually involves surgery mainly because of the fear from the complications of endoscopic therapy. In this study, clinical and radiological features of anomalous opening of CBD into the duodenal bulb as well as treatment modalities and long-term follow-up results have been evaluated. To the best of our knowledge, this is the largest series reported in the literature and the only one, which emphasizes the role of endoscopic therapy in this rare clinical entity.

## Methods

The data were obtained from the records of 12.158 patients, which had undergone ERCP at a major referral center in a 9-year period (June 1997–June 2006). The medical records of the patients who had anatomically abnormal opening of the CBD into the first part of duodenum have been collected and their transabdominal ultrasound, computerized tomography, upper endoscopy and ERCP findings have been reviewed. All the patients were followed-up on a regular basis of every 2 to 3 months and clinical and laboratory findings were assessed at the time of data collection for the outcome of the treatment modalities. In the follow up period, the number of cholangitis attacks with or without stone/sludge, the time period between attacks and treatment modalities which were applied in each admission were all extracted.

The anomalous opening of the CBD into the bulb was defined as the failure to demonstrate a papilla or papillary-like structure in its original site in the second part of the duodenum and obtaining a cholangiogram via giving a contrast medium into an orifice in the bulb during ERCP. (A previous choledocoduodenostomy operation must be excluded) In the case that the diagnosis was made by PTC, observing the tip emerging from the orifice in the bulb upon placement of the catheter into the CBD and failure to demonstrate an entry into a more distal orifice during cholangiogram were defined as an anomalous opening into the bulb.

ERCP findings were evaluated in regards to the dilatation or presence of air in the biliary tract, the configuration of CBD, the presence of stone/sludge or stricture and to the appearance of pancreatic duct in the case, it could be visualized. Presence of a stricture at the distal end of CBD was defined as a *Real Stricture *if an 11.5 mm biliary stone extraction balloon could not pulled out easily from the choledochus in case of not presenting a stone in CBD.

The diagnosis of cholangitis has been made in the presence of fever/chills, jaundice, right upper quadrant pain, leukocytosis and elevated cholestatic enzymes. The presence of three of the following features on follow-up has been accepted as symptoms of recurrent stone disease: biliary colic, jaundice and/or elevated cholestatic enzymes as well as the demonstration of bile duct dilatation on imaging.

The existence of gastric and/or duodenal ulcer, duodenal deformity or stenosis as well as the entrance of pancreatic canal has been noted.

The study was approved by the Local Ethics Committee of Yuksek Ihtisas Hospital and a written consent was also obtained from all the patients for the publication of data and figures.

## Results

The opening of the CBD into the duodenal bulb was detected in 53 cases (0.43% of total). There was a striking male preponderance (49 male, 4 female). Median age of the group was 55 years (range, 36–78). Demographical data and ERCP finding were available in all but clinical characteristics and laboratory findings could be evaluated in 39 patients only.

### Clinical characteristics (Table 1)

**Table 1:** The characteristics of patients with anomalous opening of the common bile duct into the duodenal bulb

***Demographic Data***	***Number of total cases n = 53(%)***
Median age (y)	55 (36–78)
M:F	49:4

***Clinical Characteristics***	***Available in 39 patients***

Symptoms	
Biliary Pain	37 (95)
Without (biliary) symptom	2 (5)
Fever/chills	23 (59)
Laboratory findings	
Elevated ALP-GGT	38 (98)
Jaundice	30 (77)
Leukocytosis	23 (59)
Medical History	
Cholecystectomy	25 (64)
Gallbladder stone	*22*
Acalculous cholecystitis	*2*
Acute cholecystitis with stone	*1*
Recurrent Cholangitis	10 (26)
Recurrent Duodenal Ulcer	24 (45)
Gastroenterostomy due to duodenal Stenosis	8 (21)
Concomitant diseases at diagnosis	
CBD stone	27 (51)
GB stone	8 (21)
Duodenal ulcer	8 (21)
Gastric ulcer	1 (2.5)
Liver abscess	1 (2.5)

On evaluation of the 39 patients with clinical and laboratory data, 37 (95%) had episodic abdominal pain, and 2 had no biliary symptom (ERCP was performed for unexplained cholestatic enzyme elevation in one case and for definite diagnosis in the other case who had chronic diarrhea and oozing of bile from an orifice in the bulb was seen on upper endoscopy). Twenty-three patients (59%) presented with cholangitis and one of them had sub diaphragmatic abscesses as well.

### Laboratory features

Leukocytosis (WBC > 10.000/mm3) was detected in 23 (59%) cases. Hyperbilirubinemia (total bilirubin > 1.2 mg/dL) was evident in 30 patients (76.9%) and elevated levels of Alkaline Phosphatase and Gamma Glutamyl Transpeptidase (ALP > 230 IU/L, GGT > 48 IU/L) were found in all cases except one (97.4%) who was diagnosed incidentally during the upper gastrointestinal endoscopy.

### Past medical history

25 patients (64.1%) had cholecystectomy (Due to gall bladder stone in 22 whose 4 had concurrent CBD stone which was also extracted during operation, due to acute cholcystitis in 3) Two of them had acalculous cholecystitis. One patient had retroperitoneal perforation during a stone removal from the CBD. Recurrent cholangitis was evident in 10 patients (25.6%). Recurrent duodenal ulcer was found in 24 (61.5%). 8 subjects (20.5%) had undergone gastric bypass operation due to gastric outlet obstruction related to peptic ulcer in the past.

### Indications of ERCP

CBD stone was the most common indication (49 patients, 92.5%). In two cases, ERCP was performed following a CBD injury during cholecystectomy and in the remaining 2 cases for definitive diagnosis of pancreatic cancer and to investigate biliary tract anatomy respectively (the one who had bile coming out from an orifice in the bulb during upper endoscopy).

### Endoscopic findings

**Figure 1:**
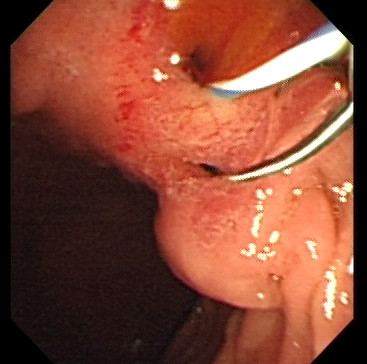
**Endosopic view of the opening site of CBD in the duodenal bulb**. Slit – like ectopic openings of common bile duct (upper hole) and pancreatic duct (lower hole) with guide-wires inserted for the better demonstration.

**Figure 2:**
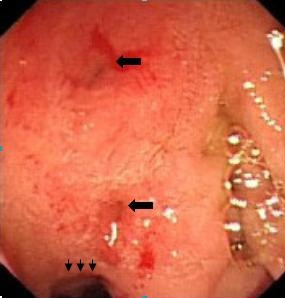
**View of deformed bulbus due to chronic ulceration**. Wall deformity and narrowing of the lumen (arrow heads). Arrows indicate openings of CBD (upper) and PD (lower).

Excessive bile and/or bile precipitates in the gastric lumen was realized in all patients with ectopic opening of the CBD into the duodenal bulb. Both the site and appearance of the choledochal entry in the bulb were unusual anatomically looking quite different from the major duodenal papilla. It was frequently located in the posterior wall of the bulb or close to the apex, slightly elevated from the mucosal surface and in some cases, like a cleft on level with the surface. In all visualized cases, pancreatic duct was opening separately next to the biliary orifice and was in a similar anatomic appearance.

(Figure 1, see also Additional file 1) There was a deformity and stenosis in the bulb secondary to past ulcers in 26 subjects (49%) with an active duodenal ulcer in 8 of them.(Figure 2) In another 8 patients, anastomosis of a prior gastroenterostomy was observed. Gastric ulcer was seen in a single patient.

### Cholangiographic findings (Table 2)

**Table 2:** Cholangiographic findings of patients

**Cholangiographic findings**	**No. of cases (n = 53) (%)**
Hook-shaped and tapered ending of CBD	53 (100)

Dilated CBD (> 10 mm)	50 (94)
with stone	*27 (51)*
without stone	*23 (43)*
Non-dilated CBD	3 (6)
Real stricture at the distal end	2 (4)
Pneumobilia	11 (21)
Pancreatic duct visualization	12 (22,6)
Dilated pancreatic duct	4 (33)

**Figure 3:**
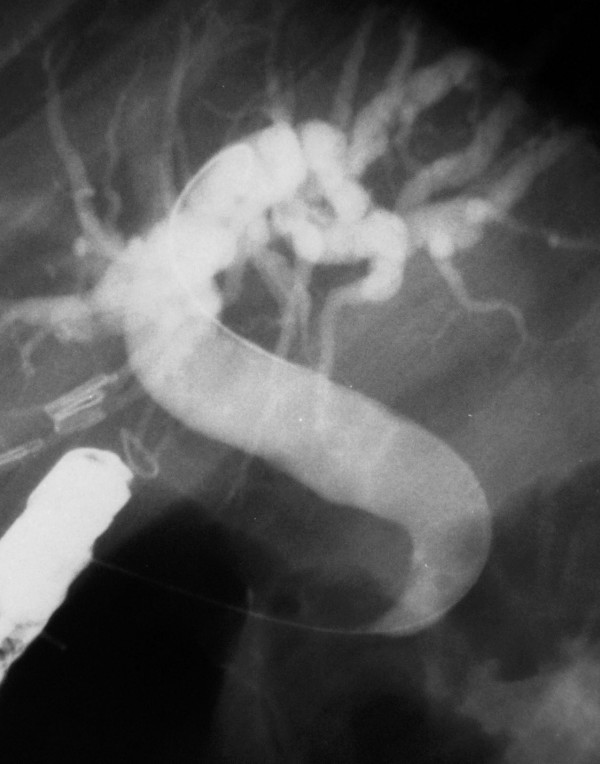
**Cholangiography of a typical case**. Characteristic appearance of CBD with tapered and hook-shaped distal ending in a patient with ectopic opening to the duodenal bulb. Note the dilation at CBD and intrahepatic bile ducts.

**Figure 4:**
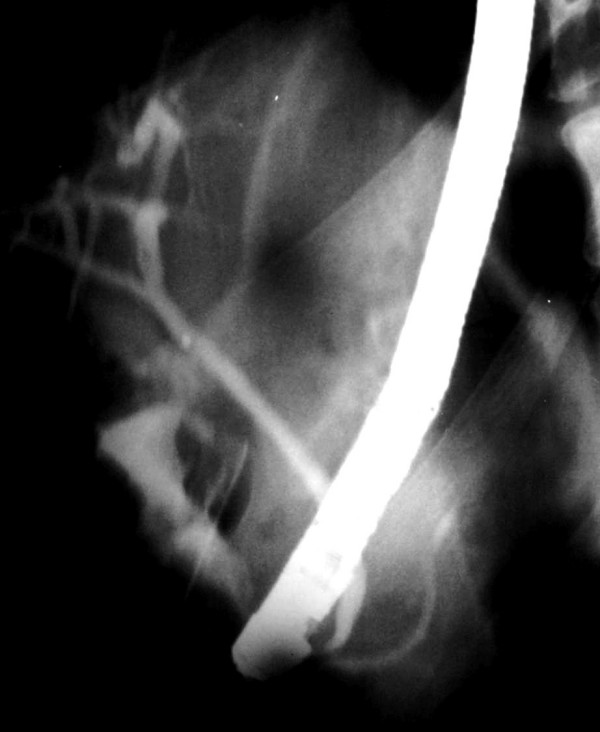
**Cholangiography of a case without dilation in CBD**. CBD and PD in normal diameter entering to the duodenal bulb separately. The hook – shape and tapering of the distal end of the CBD is now slightly perceived when it's in normal diameter

**Figure 5:**
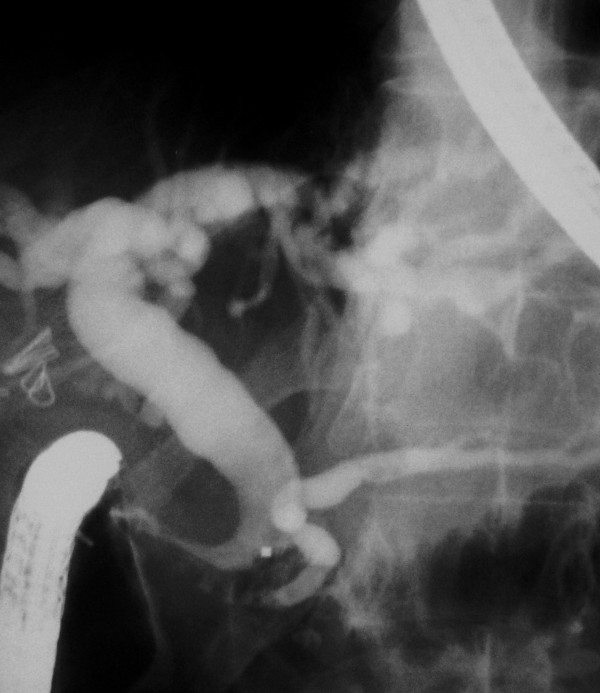
**Cholangiography of a case with "Real Stricture"**. A dilated CBD and PD separately opened to the duodenal bulb. Note the distal end of the CBD narrowed critically. Despite the absence of a stone, a 11.5 mm diameter inflated extraction balloon wasn't pulled-out.(real stricture).

Cholangiographic findings were evaluated in all 53 cases. The diagnosis was made by ERCP in a total of 51 patients (96%) and in the remaining two by PTC. As an interesting finding that was observed on cholangiography of all the patients, common bile duct was hook-shaped and tapered at the distal part that opens to the duodenal bulb (Figure 3). The CBD was dilated (>10 mm) in 50 patients (94%) and both CBD and intrahepatic bile ducts (IHBD) were of normal size in the remaining 3 (6%) (Figure 4). PTC showed dilated CBD in 2 cases in whom cannulation of CBD during ERCP was not successful due to the deformity of the bulb. Stones with dilatation in CBD were demonstrated in 27 patients (51%), but none was found in 23 (43%) who had CBD dilatation on cholangiogram. In addition, biliary leakage following a complicated laparoscopic cholecystectomy was detected in two cases. Pancreatic duct was dilated in 4 out of 12 (33%) cases who had an available pancreatogram. (Figure 5) None of them had clinical findings of pancreatitis. Air in the IHBD was detected in 11 (21.5%) patients on fluoroscopic examination before ERCP (pneomobilia or air cholangiogram).

### Other imaging studies

**Figure 6:**
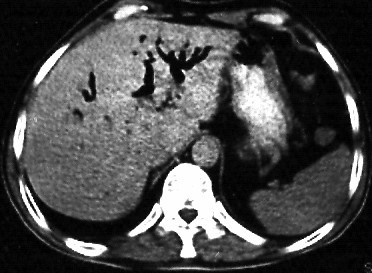
**Pneumobilia**. Computarized Tomography appearance of air in the intrahepatic bile ducts.

All the patients had an abdominal ultrasound imaging. Fourteen (35.9%) had air in the IHBD on transabdominal ultrasound. The CBD and/or IHBD dilation and CBD and/or gall bladder stones were the other frequent findings on ultrasound evaluation. Liver abscesses were detected in one patient by ultrasound also. In 3 (7.7%) patients who had upper gastrointestinal series for abdominal pain, barium reflux into the IHBD was detected. CT was performed in only two patients and findings were nonspecific for ectopic CBD opening into the duodenal bulb except one who had air in the IHBD considering the possibility of abnormality. (Figure 6).

### Treatment (Table 3)

**Table 3:** The summary of therapeutic approach to the patients with anomalous opening of the CBD into duodenal bulb

Cholangiographic Findings	Endoscopic treatment	Surgery	Medical treatment	No treatment	**Total (n = 53)**
**Dilated CBD**					**50**
with stone	19 (%70)	8 (%30)	-	-	**27**
without stone	7 (%30)	-	16 (%70)	-	**23**
**CBD w.o dilation**	2*	-	-	1**	**3**
**Total **(n = 53)	**28 **(%53)	**8 **(%15)	**16 **(%30)	**1 **(%2)	**53 **(%100)

* Patients with bile leakage after cholecystectomy (treatment with nasobiliary drainage alone)

** The patient diagnosed accidently during upper GI endoscopy and without biliary symptom

Of the 50 patients with a dilated CBD, 26 (52%) patients were treated endoscopically, 16 (30%) patients recovered with medical treatment (antibiotics, intravenous fluid replacement, etc.) and 8 (16%) underwent surgery. Two patients (2/53) with ectopic opening of the CBD detected incidentally during ERCP performed for biliary leakage after laparoscopic cholecystectomy were also treated endoscopically. No intervention was done in a single case who had a normal CBD and diagnosed incidentally during a work-up for diarrhea. Balloon dilatation of the duodenal bulb was required in a total of 12 cases (22.6%) before the cannulation of the CBD.

**Figure 7:**
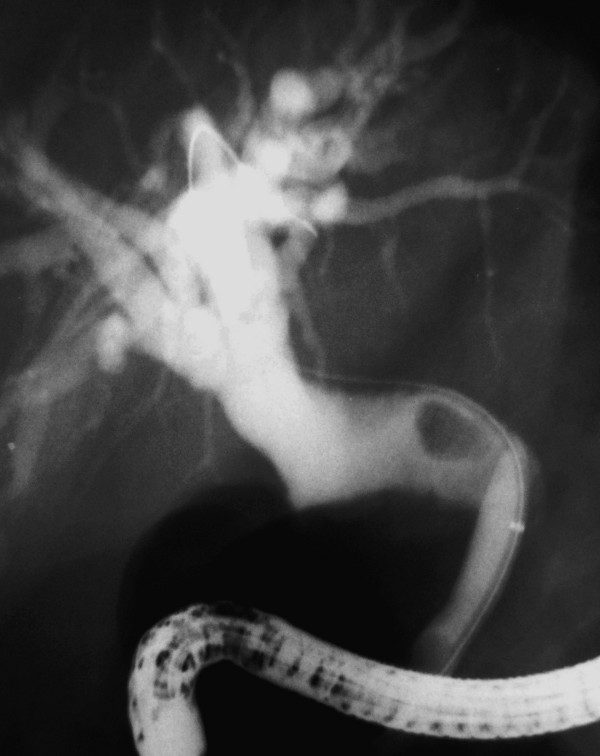
**Endoscopic Papillary Balloon Dilation**. Dilation of the ectopic opening with a 8 mm diameter biliary dilation balloon. Note the stone next to the balloon.

In twenty-seven patients with stone in the CBD, only 8 (30%) in whom endoscopic treatment failed due to the stones larger than 2 cm had to undergo surgery. The remaining 19 patients (70.4%) were treated endoscopically as follows:

Endoscopic sphincterotomy (ES) was performed in only two cases. In the remaining 14 (73.5%) stone was extracted using balloon or dormia basket following the dilatation of the papilla by 8-mm biliary balloons or up to 15-mm pyloric dilatation balloons for larger stones (figure 7). Stenting was the sole treatment in three patients who had a stone more than 2 cm in diameter and had significant co-morbidities with advanced age. Endoscopic stone extraction was successful in 16 of 27 cases (59%).

In 23 patients who had CBD dilatation without stone or sludge, CBD was swept by balloon during ERCP to confirm the absence of stone or sludge. This method was also used to decide whether the narrowing of the distal end of the CBD was critical. When the 11.5 mm diameter stone extraction balloon was not extracted easily, we concluded that a real stricture exists. Plastic stent was placed in 2 who had critical distal stenosis, which was thought to impede drainage. In 5 patients, NBD catheter was placed to relieve cholangitis.

### Complications

They occurred in two cases as bleeding and perforation both following EST. Bleeding stopped spontaneously and perforation was treated medically. Balloon dilatations were performed in both the pylorus and papilla without any complication.

### Follow-up (Table 4)

**Table 4:** The summary of seven patients with the recurrence of initial presentations during follow up period

**Clinical presentatiton**	**No. of patients**	**No. of episods**	Time* (median/month)	**Treatment**
Cholangitis	**5**	**9**	**19.5 (14–47)**	Medical
-without real stricture	3	3	28.3	Medical
-with real stricture	2	6	15.5	Endoscopic (dilatation and/or stenting)
CBD stone	**2**	**2**	**21.5 (16–27)**	Endoscopic (stone extraction)
**Total**	**7**	**11**		

*Time from initial treatment to reccurence

During a mean follow-up of 31.2 ± 27.9 months (range, 1–90 months), seven patients (all male, age range 36–65, median 49 years) had recurrent symptoms due to recurrent episodes of cholangitis in 5 and CBD stone or sludge in 2. The recurring pathology was the same as the original one. In five patients who presented with recurrent cholangitis, nine episodes were observed totally (range, 1–3 episodes per patient). The time to the recurrence of cholangitis attack in three who had a single episode during the follow-up period was 16, 22 and 47 months respectively (mean, 28.3 months). In the remaining two who experienced three episodes each, the time to recurrence was ranging between 14 to 17 months (mean, 15.5 months). In two patients with recurrent CBD stone or sludge; only a single episode for each was detected. In these two cases, the time from the extraction of the first stone/sludge to the diagnosis of the recurrence was 16 and 27 months (mean, 21.5 month).

The time to recurrence of symptoms following diagnosis of CBD stone and cholangitis was ranging from a minimum of 14 to 16 months to a maximum of 27 to 47 months respectively. Stone removal with balloon and/or basket was performed in two patients who had recurrent stone disease. In addition, balloon dilatation of the duodenal bulb was required to reach the orifice in these two patients. In two patients who had a real stricture in the distal CBD and recurrence of cholangitis, biliary dilatations with 5–8 mm dilatation balloons and/or plastic stenting were performed on each session. The remaining three patients who had recurrent cholangitis episodes recovered with only medical therapy.

Except those patients with recurrent symptoms, all the remaining patients confirmed on phone calls that they were not admitted to another hospital because of biliary symptoms. There was no hepatobiliary malignant disease or death in any patient during the follow-up period

## Discussion

The most outstanding feature of the normal anatomy of the extrahepatic biliary system is its high degree of variability. In the rare studies about this subject, abnormal opening of the common bile duct (CBD) is reported at a wide range of 5.6 to 23% due to the limited number of cases [[1,5] and [6]]. Lindner et al. examined 1,000 intraoperative cholangiograms and they found that the rate of distal opening versus the normal anatomic site was 13.1%; however, no proximal opening was indicated [7]. As can be seen, the distal opening is not rare, but a proximal opening, especially into the duodenal bulb, is reported rarely, consisting of only a few cases [8]. Lee et al. recently reported a large series concerning clinical implications of this abnormality [9].

The etiology of this anomalous opening is not known, but it has been argued that developmental errors formed during embryogenesis, which are not yet understood, could be a causative factor. The liver originates from the hepatic diverticulum, the cranial part (pars hepatica) of which gives rise to the intrahepatic and common hepatic ducts and the caudal part (the pars cystica) gives rise to the gall bladder and the cystic duct [10]. The most commonly adopted hypothesis about the ectopic opening of the CBD was proposed by Boyden [8], who explained that the ectopic CBD opening was formed by coincidental subdivision of the primary hepatic furrow, as it becomes pars hepatica and pars cystica. He summarized the process as follows; "In the first weeks of embryogenesis, if subdivision occurs very early, leaving the pars hepatica above the zone of growth that separates stomach from duodenum, then the pars hepatica will develop into a duct emptying into the region of the pylorus".

To date, in those cases with ectopic opening of CBD into the duodenal bulb, the relation of embryonal defects with sex has not been discussed in the published literature, but evident male predominance is remarkable according to case reports. In one series, fifteen of 18 patients, and in another series, seven of eight patients were male [9,11]. In the presented series, the male to female (m/f) ratio was 49/4. When the ectopic openings of CBD to the other sites of the digestive tract were evaluated, there was no significant difference according to sex, whereas male predominance in patients with ectopic opening to the duodenal bulb is indicative, and warrants explanation.

Although the age range of patients in this study was between 36–78 years, the median age at diagnosis was 55. The most frequent symptom was biliary pain and was present in 95% of patients during admission. However, since the diagnosis was made in their fifth decade, it is obvious that some of the patients were asymptomatic for a long period. Indeed, two patients (5%) in this series were diagnosed coincidentally. One of these patients was 78 years old and diagnosed during the etiological search of elevated cholestatic enzyme levels; another patient had been evaluated for diarrhea.

When the past medical history of some of these patients was questioned, a history of cholecystectomy (64%) and/or ulcer (20.5%) operations because of biliary and duodenal diseases was revealed in a significant number of patients, as well as of medical treatment due to recurring duodenal ulcer (61.4%) and/or frequent cholangitis attacks.

In fact, diseases related with the biliary system are reported frequently in patients with ectopic CBD opening[2-4,9,11-18]. In this study, CBD dilatation was found in 94% of the cases with or without intrahepatic biliary dilatation. Additionally, 51% of the patients had CBD stone and 59% of the patients had cholangitis attacks. Liver function tests were abnormal in 97.4% of the patients, and 76.9% had evident jaundice. In our opinion, duodenal mucosal diseases (ulcer and deformation/stenosis) and the biliary system diseases are strongly related with the site and structure of the ectopic opening. In the literature, histological examination of double CBD cases with openings into the stomach demonstrated that there was no sphincteric structure at the site of the openings [19-22].

In a presentation with two cases, one of the cases who had an anomalous opening at the third part of the duodenum had intact sphincteric function manometrically, whereas there was no sphinteric function in the other case with opening into the duodenal bulb[23]. In all of our cases, there was a loose, cleft-like opening instead of a normal papillary structure, through which bile could easily flow into the duodenal bulb.

This situation causes a two-sided interaction between the duodenum and biliary tract:

Duodenal content will pass through the biliary tract easily (duodenobiliary reflux) and Bile can flow into the duodenal bulb without sphincteric control. In our opinion, the first of these actions causes biliary tract diseases and the second causes ulcer and complications. Duodenobiliary reflux was examined in the literature. Bacterial contamination continues for months even years in approximately 30–70% of patients after endoscopic or surgical sphincteroplasty [24,25]. However, occurrence of cholangitis together with sphincteroplasty is rare, and if the orifice is open, the rate is less than 3% [26]. There are, however, cases reported in the literature with recurrent cholangitis attacks after choledochoduodenostomy[27] and endocopic sphincterotomy (EST)[28]. It is proposed that duodenobiliary reflux and temporary obstructions cause the cholangitis attacks without restenosis. This idea is supported by evidences like barium or air reflux (pneumobilia) through the biliary tract [29] and extraction of food debris in some cases from the CBD[25].

In our series, air was seen in the intrahepatic bile ducts in 35.8% of patients by transabdominal USG, in 28% of patients fluoroscopically during ERCP and in two of the patients by CT. Barium passage through the biliary tract was indicated in three patients in whom upper gastrointestinal tract barium series were taken, and food debris was extracted from the pancreatic duct in one case. Similar findings were reported in the literature in patients with ectopic bilioenteric openings [[2,3], and [30]]. Although not demonstrated to date, there is possibly bacteriobilia in those cases that would not cause any problem as long as bile flux continues just like in sphincterotomy or choledochoduodenostomy cases. It is possible that duodenal content refluxing into the biliary system would cause temporary obstructions at times that might lead to bacterial growth triggering cholangitis attacks. The improvement in more than two-thirds of our patients with dilated CBD but without stone with only medical treatment supports this idea. Furthermore, temporary obstruction at the biliary opening site due to edema in the duodenal bulb caused by chronic ulceration and/or inflammation is another possibility in many patients. Bacterial overgrowth resulting from stasis in the biliary tract due to temporary obstructions or distal stricture is likely to cause the bile to gain lithogenic characteristics. Moreover, the reflux material itself also causes stone formation. It has been reported in the literature that medical materials like sutures [31] and metalic clips [32], vegetable debris such as cherry stalk [33] and tomato skin [34], and foods like fish bones [35] form a nidus for biliary stone formation.

The second most common finding in these patients was ulcer disease and its complications like deformation and stenosis. Duodenal bulb deformation was found in 49% of the cases. There was active duodenal or gastric ulceration in 31% and 1.8% of these patients, respectively. Eight cases (31%) had a gastric by-pass operation because of duodenal obstruction. As mentioned above, the second result of the absence of a sphincteric structure in the ectopic biliary opening site is the free bile flux into the duodenal bulb and stomach without control. Bile is very ionic and can lead to mucosal damage by deteriorating the surfactant characteristics of the protective mucous layer [36]. Deformation of the duodenal bulb can develop after chronic exposure to the noxious effects of ionic bile acids and alkaline pH causing mucosal damage and ulceration, which cannot be prevented by cytoprotective mechanisms. Similar effects might also occur in gastric mucosa with the duodenal content's passage through the stomach[37]. In some patients, opening of the pancreatic duct into the duodenal bulb was seen as a loose cleft, similar to the CBD opening. Similar to the bile, flowing of the pancreatic fluid that has a high pH and contains proteolytic enzymes and bicarbonate may contribute to ulcer formation. Furthermore, these patients had significantly higher cholelithiasis (28%) and cholecystectomy (64%) rate. It is known that adding one of those two situations increases the duodenogastric reflux caused by biliary sphincter ablation [38]. A similar situation might occur in these cases.

Although the bulbus was deformed in half of the cases, ERCP success rate in diagnosis was 96%. Cannulation was not possible in only two (4%) patients due to advanced deformity in the bulbus, and these patients were diagnosed by PTC. In all cases, the CBD made a sharp curve and was tapered at the distal end entering the duodenal bulb at a right angle (Figure 3). This finding is called "hook or sickle-shaped" in the literature and was seen in all of our cases. In our opinion, the tapered end of the CBD is due to the sharp right angle on its way to the duodenal bulb. It is a relative tapering when compared to the proximal part of the CBD and does not mean "real stricture". The authors of this study use the phrase "real stricture" because the tapered distal end which is a characteristic feature of these patients, together with the dilated proximal bile ducts, creates a stricture-like appearance caused by the relative diameter difference of the proximal and distal ends. We pulled out an 11.5 mm stone extraction balloon in each case to control whether a stone was present. When the balloon could not be extracted easily, in the case of there is no stone; we accepted that these patients had a "real stenosis"(Figure 5). It is not known why a stricture develops at the distal end of CBD. Lee et al. found fibrofatty proliferation in the peribulbar area on CT in three of eight patients with ectopic biliary openings [11]. Although the authors made no comment in their study, the increase in the reactive connective tissue around the bulb was possibly due to duodenal ulceration and deformation. We conclude that it can be a finding that can explain "real stricture" formation in the opening site including the distal end of the CBD in some cases. In contrast to the literature, the distal stenosis rate in our series was very low (two cases), probably due to our different definition of the situation.

The pancreatic duct, which was visualized in 12 cases, had an opening similar to the CBD's cleft-like opening, and opened into the duodenal bulb 1 to 2 cm next to it. (Figure 2 and 3) Pancreatic duct dilatation was found in four cases (33%), but none of these patients had any history or finding of pancreatic disease. In the literature, there is limited data about the pancreatic duct in those patients. Lee et al. visualized the pancreatic channel in seven patients. All of the patients had pancreatic channel openings into the duodenal bulb; channel dilatation was seen in two patients, but it was not clarified whether the CBD opening was together with the pancreatic opening [9]. In another large series, the pancreatic canal was visualized in one of the eight patients and was opened to the papillary orifice in the bulbus, which also appeared normal [11]. The pancreatic channels of 11 patients who had CBD opening into the duodenal bulb were visualized in the literature [4,9,11,15]. Acute pancreatitis was diagnosed in only one case (there was no finding supporting pancreatitis other than elevated amylase level in this case, who was also a heavy alcohol user) [15]. Adding our 12 cases to the literature, there are 23 cases in total, and pancreatic duct dilatation was found in a total of six (26%) patients. This may be due to possible obstructions in the pancreatic channel in some cases from time to time. There is a great possibility of reflux of the duodenal content into the pancreatic duct, because the structure of the pancreatic opening in our patients was very loose, without a sphincter, and even food debris could easily reflux into the canal.

Although it is a rare situation, since most of the patients have deformity and stenosis in the duodenal bulb, anomalous CBD opening into the duodenal bulb should be considered in intractable and recurrent ulcer cases. Surgeons are cautioned to use extreme care during the surgery of peptic ulcer disease in order to avoid biliary injury, which can be fatal[39,40]. The surgeons in our eight cases with peptic ulcer operation must have exerted extreme care because they performed only gastric by-pass operation (simple gastroenterostomy) and not operations like Billroth I or II, which could damage the biliary system. Although the patients were not diagnosed at that time, the surgeons no doubt realized the anomaly, possibly during the releasing of the duodenum (Kocher maneuver), and abandoned the decision to apply the Billroth procedure.

Choledochoduodenal fistulas, surgical or spontaneous, which also cause free bile flux from an orifice into the duodenum, must be considered in the differential diagnosis.

Biliodigestive fistulas are rare and 70% of these are choledochoduodenal fistulas [41]. Those fistulas can develop in only choledocholithiasis without a history of any biliary surgery, ERCP, pancreatitis or ulcer [42]. Especially the proximal type of choledochoduodenal fistulas, which have a relation with the duodenum from the more proximal to the distal 2 cm end of the CBD, can be confused with anomalous biliary opening. This situation might occur due to fistulization of duodenal ulcer to the CBD [43], and can cause an endoscopic appearance similar to ectopic opening anomaly with ulcer. A distinction can easily be made in these cases by seeing normal papilla in the second part of the duodenum distal to the orifice and by imaging of the more distal part of the CBD to the catheter entry point when the contrast material is delivered from the orifice.

Periampullary tumors must also be differentiated because the dilatation of the pancreatic duct and CBD together, and a sudden ending of the CBD, are common features of the anomalous opening of CBD and of the periampullary tumor (pancreatic carcinoma must also be differentiated). In our series, one of the patients who was referred as periampullary tumor due to dilatation of CBD and pancreatic duct was diagnosed as anomalous opening, and there was food debris in the pancreatic duct. The patient was also evaluated by different visualization techniques and no malignancy was found. During the follow-up period, there was no malignancy development. In the literature, two cases with anomalous opening of the CBD to the duodenal bulb had pancreatoduodenectomy operation for the suspicion of periampular tumor. Afterwards it was reported that there was no tumoral mass in the specimens[9,11].

As to the therapy, surgery is the main treatment modality in most of the case reports in the literature. Choledochoenterostomy, surgical stone extraction by choledochotomy and rarely, pancreatoduodenectomy are the surgical procedures reported thus far. Lee et al. preferred surgical treatment in 13 (72%) of 18 patients [9]. In another series, seven of eight patients (87.5%) underwent surgical treatment [11]. Primary endoscopic treatment is reported rarely [14]. There are two cases in the literature that improved spontaneously or by supportive medical treatment without any invasive procedure[11,15].

In the presented series, endoscopic approach was our primary diagnostic and therapeutic modality. ERCP was performed successfully in 96% (51/53) of the cases and 52% (26/50) of the patients with a dilated CBD (with or without stone) was treated endoscopically. Stone extraction was successful in 59% (16/27) of the patients with stone in CBD. In the vast majority (14/16; %87.5), it is performed by papillary baloon dilatation. Stenting was preferred in three (11%) cases with stones due to advanced age and comorbidities. The total endoscopic treatment success rate was 70% (19/27) in the patients with CBD stone. Surgery was considered in only 16% (8/50) of the patients whose stones could not be retrieved endoscopically due to the size (>2 cm) with or without a real stricture distal to the CBD which has been described previously in this article. Nasobiliary drainage was another endoscopic treatment option in the cases with cholangitis and without any stone in the CBD. Interestingly, in 32% (16/50) of the patients who were symptomatic with a dilated CBD but without stone, symptoms improved via supportive medical treatment solely or spontaneously. Overall, half of the cases improved by endoscopic treatment, one-third improved by supportive treatment or healed spontaneously, and only a few patients needed surgery.

Surgery was the major treatment modality in the past because it was thought that endoscopic sphincterotomy (ES) could cause bleeding and perforation due to absence of the sphincteric structure and intramural part of the CBD, which was oblique to the duodenum. Two perforations (3.7%), one of which occurred in a different center, were seen during ES in our series. The perforation rate was higher than in ES performed on normal papilla, in which one patient had bleeding (1.8%). After our perforation experience, during EST in these specific patient groups, we performed the Endoscopic Papillary Balloon Dilatation (EPBD) method successfully. We used "Through The Scope" (TTS) biliary dilatation balloon (6–8 mm in diameter) or 10–12 mm diameter TTS pyloric dilatation balloon for larger CBD stones. The orifice was dilated using standard dilatation procedure under fluoroscopy and then CBD stones were extracted using stone

extraction balloon and/or dormia basket. It was shown that the perforation and bleeding rate of EPBD in normal papilla was lower than in ES [44,45]. In those cases with anomalous opening of the CBD into the bile duct, or in whom the sphincteric structure is absent or very weak, the procedure was not a real sphincteroplasty, but rather a dilatation procedure of the opening as far as the duodenal wall flexibility allowed. For some of the patients, this process is dilatation of a stricture that developed in the ectopic orifice over time because of mucosal edema, ulceration and duodenal deformation. Due to deformation/stenosis in the bulbus, CBD cannulation was possible in 12 patients (22.6%) only after duodenal bulb dilatation. There were no complications during bulbar and biliary orifice balloon dilatations. Two cases who suffered from perforation during EST improved with supportive treatment without any surgery, and endoscopic CBD stone extraction was carried out later. Bleeding spontaneously stopped in a patient who bled during EST, and neither blood transfusion nor endoscopic operation was needed to stop the bleeding.

During an average of 31-month of follow-up, there were no deaths due to the biliary system disease. No GIS-related cancer case was found. In the literature, only a single case of pancreatic cancer was reported among cases with double CBD dilatation [12]. In double CBD cases that open into the stomach, cancer was reported in one case, but no cancer was reported in patients whose CBD opened to the duodenal bulb[12,46].

Symptoms of seven patients recurred during follow-up (2 cases with CBD stone, 5 cases with recurrent cholangitis). The recurrence rate (13.2% of all cases), the number of attacks and period between the attacks were within acceptable limits (11 episodes in a total of 7 patients, 14–47 months, average 20 months). The minimal period for cholangitis attack in recurrent cases is 14 months, and for stone development 16 months, but it could be as long as 47 months for cholangitis attacks and as long as 27 months for stone formation. An interesting finding is that in five (71%) of seven cases, duodenal deformation was present, and in one case there was also an active duodenal ulcer. Duodenal bulb stenosis was so severe in two patients with CBD stones that balloon dilatation was needed in order to reach the biliary opening. A higher rate of duodenal damage in recurrent patients, when compared to the duodenal deformation rate in all cases (49%), supports the opinion that edema, ulceration and deformation in the duodenum are sometimes effective in causing formation of biliary pathologies in these patients. Another factor for the recurrence is the real stricture at the distal end of the CBD in two of five patients who were admitted with recurrent cholangitis attacks. In comparison to the three cases who had only one attack, those two cases with three attacks had shorter attack duration (mean 15.5 months vs. 28.3 months). This is also a determinant for the treatment approach because while the other three patients' clinical situation was improving with only supportive treatment; these two patients with stricture needed dilatation and/or stent application.

In conclusion, based on these findings, presence of duodenal deformation and/or ulcer and its severity play a significant role in the patient's long-term follow-up and the treatment strategy. The patients with distal, "real stricture" may be considered as suitable candidates for surgery. Each patient must be evaluated individually and the surgery must be decided weighing risks and benefits of the operation. Given the low recurrence rate in the long-term follow-up and acceptable period of time to recurrence, endoscopic therapy should primarily be considered in all but those patients who has "real stricture" at the end of the CBD. Surgery should be reserved for the patients in whom endoscopic treatment is failed or for those with the stricture in the distal CBD.

## Conclusion

Anomalous opening of the CBD into the duodenal bulb is a very rare anomaly, which can cause severe clinical outcomes due to its anatomic location. It can also have different characteristics from the other bilioenteric opening anomalies due to its specific localization. The indirect findings of anomalous opening in a patient who does not have bilioenteric anastomosis are biliary tract pneumobilia seen in USG, CT or direct radiographs; biliary tract barium reflux in upper intestinal barium series and finally deformed or stenosed duodenal bulb with an orifice coming out bile seen during upper GI endoscopy. In the presence of these findings, one must suspect anomalous opening of the CBD. To differentiate from the choledochoduodenal fistula, diagnosis must be confirmed by ERCP.

It is wise to remember that the treatment not only consists of operation, but also of endoscopic balloon dilatation of the orifice with stone extraction and of other endoscopic treatments such as stenting and nasobiliary drainage which can be performed successfully and safely as well. Endoscopic sphincterotomy can cause complications such as perforation and bleeding and should be avoided due to lack of a normal sphincter structure at the opening of the CBD. Surgery should only be reserved for the patients who do not respond to the endoscopic therapies with a real stricture at the end of the CBD or simply for those patients who have large stones with or without stricture.

## Competing interests

The author(s) declare that they have no competing interests.

## Authors' contributions

SD designed and carried out the study and interpreted the results.

EP contributed to organizing and interpretation of the data.

BC contributed to organizing and interpretation of the data.

CC contributed to organizing and interpretation of the data.

DO contributed to organizing and interpretation of the data.

SOD contributed to organizing and interpretation of the data.

HG contributed to organizing and interpretation of the data.

BS made the final decisions about the article before submition.

As the authors of this manuscript, we declare that we all read and approved the article before the submission.

## Pre-publication history

The pre-publication history for this paper can be accessed here:



## Supplementary Material

Additional file 1 Anomalous opening of the CBD into the duodenal bulb, cannulation of CBD and PD during ERCP. This movie shows the anomalous opening of the CBD into the duodenal bulb during cannulation. Please note separate opening of the pancreatic duct and narrowed lumen of the bulbus. A linear ulcer next to the orifices can be seen. One can also realize how difficult to find the orifices and stabilize the endoscope over there for the cannulation because of the narrowing of the lumen and position of the endoscope. Click here for file

## References

[B1] Lurje A (1937). The topography of the extrahepatic biliary passages. Ann Surg.

[B2] Doty J, Hassal E, Fonkalsrud EW (1985). Anomalous drainage of the common bile duct into the fourth portion of the duodenum. Arch Surg.

[B3] Quintana EV, Labat R (1974). Ectopic drainage of the common bile ducts. Ann Surg.

[B4] Kubota T, Fujioka T, Honda S, Suetsuna J, Matsunaga K, Terao H (1988). The papilla of Vater emptying into the duodenal bulb. Report of two cases. Jpn J Med.

[B5] Schulenberg CAR (1970). Anomalies of the biliary tract as demonstrated by operative cholangiography. Med Proc.

[B6] Keddie NC, Taylor AW, Sykes PA (1974). The termination of the common bile duct. Br J Surg.

[B7] Lindner HH, Pena VA, Ruggeri RA (1976). A clinical and anatomical study of anomalous termination of the common bile duct into the duodenum. Ann Surg.

[B8] Boyden EA (1944). Congenital variations of the extrahepatic biliary tract: a review. Minn Med.

[B9] Lee SS, Kim M-H, Lee S-K (2003). Ectopic opening of the common bile duct in the duodenal bulb: clinical implications. Gastrointest Endosc.

[B10] Tan CE, Morosco GJ (1994). The developing human biliary system at the porta hepatis level betwen 29 days and 8 weeks of gestation: a way to understanding biliary atresia. Part I. Pathol Int.

[B11] Lee HJ, Ha HK, Kim MH (1997). ERCP and CT findings of ectopic drainage of the common bile duct into the duodenal bulb. Am J Roentgenol.

[B12] Yamashita K, Oka Y, Urakami A (2002). Double common bile duct: a case report and a review of the Japanese literature. Surgery.

[B13] Bernard P, Le Borgne J, Dupas B (2001). Double common bile duct with ectopic drainage into the stomach. Case report and review of the literature. Surg Radiol Anat.

[B14] Özaslan E, Saritas U, Tatar G (2003). Ectopic drainage of the common bile duct into the duodenal bulb: report of two cases. Endoscopy.

[B15] Rosario MT, Neves CP, Ferreira AF (1990). Ectopic papilla of Vater. Gastrointest Endosc.

[B16] Teilum D (1986). Double common bile duct. Case report and review. Endoscopy.

[B17] Harada A, Yokoyama Y, Yokoyama I (1983). Two cases of duplication of the common bile duct, emptying into the stomach and duodenum. Jpn J Gastroeterol Surg.

[B18] Ihara K, Ooue M, Takami K (1989). A case of double choledochus with intrahepatic stones and stenosis of the left hepatic duct. Jpn J Gastroenterol Surg.

[B19] Everett C, Macumber HE (1942). Anomalous distribution of the extrahepatic biliary ducts. Ann Surg.

[B20] Kubik S, Groscurth P (1977). Eine seltene Anomalie der extrahepatischen Gallenwege und v coronaria ventriculi. Chirurg.

[B21] Becker HD, Kempmann G, Stempfle B (1980). Akzesorricher ductus choledochus. Leber, Magen, Darm 4.

[B22] Ohkawa H, Sawaguchi S, Yamazaki Y (1982). Experimental analysis of the ill effect of anomalous pancreatobiliary ductal union. J pediatr Surg.

[B23] Foulon JP, Hardy JM, Batellier JL (1980). Ectopic openings of the common bile duct. A contribution to the physiological study of the sphincter of oddi. J Chir.

[B24] Gregg JA, Girolami PD, Carr-Locke DL (1985). Effects of sphincteroplasty and endoscopic sphincterotomy on the bacteriologic characteristics of the common bile duct. Am J Surg.

[B25] Cotton PB (1984). Endsocopic management of bile duct stones (apples and oranges). Gut.

[B26] Feretis CB, Contou CT, Manouras AJ (1984). Long-term consequences of bacterial colonization of the biliary tract after choledochostomy. Surg Gynecol Obstet.

[B27] Goldman LD, Steer ML, Silen W (1983). Recurrent cholangitis after biliary surgery. Am J Surg.

[B28] Thomas CG, Nicholson CP, Owen J (1971). Effectiveness of choledochoduodenostomy and transduodenal sphincterotomy in the treatment of benign obstruction of the common duct. Ann Surg.

[B29] Bordas JM, Elizalde I, Llach J (1996). Biliary reflux due to sphincter of oddi ablation: a new pathogenetic explanation for long-term major biliary symptoms after endoscopic – sphincterotomy. Endoscopy.

[B30] Kanematsu M, Imaeda T, Seki M (1992). Accessory bile duct draining into the stomach: case report and review. Gastrointest Radiol.

[B31] Smoczynski M, Mittlener S (1995). Suture material as a nidus of common bile duct calculi. Endoscopy.

[B32] Angel R, Abisambra N, Marin JC (2004). Clip choledocholithiasis after laparoscopic cholecystectomy. Endoscopy.

[B33] Plath F, Brock P, Hasse N (2002). Vegetable stalk as a nidus for gallstone formation in a patient with a juxtapapillary duodenal diverticulum. Gastrointest Endosc.

[B34] Szanto I, Gamal EM, Banai J (1994). Common bile duct stone formation induced by tomato skin following endoscopic sphincterotomy. Endoscopy.

[B35] Kaji H, Asano N, Tamura H (2004). Common bile duct stone caused by a fish bone: report of a case. Surg Today.

[B36] Northfield TC, Mc Coll I (1973). Postprandial concentrations of free and conjugated bile acids down the length of the normal human small intestine. Gut.

[B37] Brown TH, Walton G, Cheadle WG (1989). The alkaline shift in gastric pH after cholecystectomi. Am J Surg.

[B38] Gregg A, Carr-Locke DL (1084). Endoscopic pancreatic and biliary manometry in pancreatic, biliary and papillary disease, and after endoscopic sphincterotomy and surgical sphincteroplasty. Gut.

[B39] Roe CF, Gazzangia A, McNamara J (1963). Aberrant intrapancreatic ducts leading to fatality after gastrectomy. Am J Surg.

[B40] Dowdy GS, Waldron GW, Brown WG (1962). Surgical anatomy of the pancreatico-biliary ductal system-observations. Arch Surg.

[B41] Hess W, Demling I (1984). Cholezystitis und ihre Komplikationen. Gastroenterologie.

[B42] Karincaoglu M, Yildirim B, Kantarceken B (2003). Association of peripapillary fistula with common bile duct stones and cholangitis. ANZ J Surg.

[B43] Shimao K, Yamue H, Nishimoto N (1999). Choledocoduodenal fistula at the anterior wall of the duodenal bulb: a rare complication of duodenal ulcer. Hepatogastroenterology.

[B44] Bergman JJ, Rauws EA, Fockens P (1997). Randomized trial endoscopic balloon dilatation versus endoscopic sphincterotomy for removal of bile duct stones. Lancet.

[B45] Komatsu Y, Kawabe T, Toda N (1998). Endoscopic papillary balloon dilation for the management of common bile duct stones: experience of 226 cases. Endoscopy.

[B46] Kondo K, Yokoyama I, Yokoyama Y Two cases of gastric cancer-bearing double choledochus with ectopic drainage into the stomach. Cancer.

